# Tracking implementation strategies in the randomized rollout of a Veterans Affairs national opioid risk management initiative

**DOI:** 10.1186/s13012-020-01005-y

**Published:** 2020-06-23

**Authors:** Shari S. Rogal, Matthew Chinman, Walid F. Gellad, Maria K. Mor, Hongwei Zhang, Sharon A. McCarthy, Genna T. Mauro, Jennifer A. Hale, Eleanor T. Lewis, Elizabeth M. Oliva, Jodie A. Trafton, Vera Yakovchenko, Adam J. Gordon, Leslie R. M. Hausmann

**Affiliations:** 1 Center for Health Equity Research and Promotion, VA Pittsburgh Healthcare System, Pittsburgh, PA USA; 2 Department of Surgery, University of Pittsburgh School of Medicine, Pittsburgh, PA USA; 3 Department of Medicine, Division of Gastroenterology, Hepatology, and Nutrition, University of Pittsburgh School of Medicine, Pittsburgh, PA USA; 4 Veterans Integrated Service Network 4 Mental Illness Research, Education and Clinical Center, VA Pittsburgh Healthcare System, Pittsburgh, PA USA; 5 RAND Corporation, Pittsburgh, PA USA; 6 Division of General Internal Medicine, University of Pittsburgh School of Medicine, Pittsburgh, PA USA; 7 Department of Biostatistics, University of Pittsburgh Graduate School of Public Health, Pittsburgh, PA USA; 8 VA Office of Mental Health and Suicide Prevention, VA Palo Alto Healthcare System, Menlo Park, CA USA; 9 VA Center for Innovation to Implementation, VA Palo Alto Healthcare System, Menlo Park, CA USA; 10 Department of Psychiatry and Behavioral Sciences, Stanford University School of Medicine, Palo Alto, CA USA; 11 Center for Healthcare Organization & Implementation Research, Edith Nourse Rogers Memorial Veterans Hospital, Bedford, MA USA; 12 Program for Addiction Research, Clinical Care, Knowledge, and Advocacy, University of Utah School of Medicine, Salt Lake City, UT USA; 13 Informatics, Decision-Enhancement, and Analytic Sciences Center, VA Salt Lake City Health Care System, Salt Lake City, UT USA

**Keywords:** Tailoring, Evaluation, Adaptation, Monitoring

## Abstract

**Background:**

In 2018, the Department of Veterans Affairs (VA) issued Notice 2018-08 requiring facilities to complete “case reviews” for Veterans identified in the Stratification Tool for Opioid Risk Mitigation (STORM) dashboard as high risk for adverse outcomes among patients prescribed opioids. Half of the facilities were randomly assigned to a Notice version including additional oversight. We evaluated implementation strategies used, whether strategies differed by randomization arm, and which strategies were associated with case review completion rates.

**Methods:**

Facility points of contact completed a survey assessing their facility’s use of 68 implementation strategies based on the Expert Recommendations for Implementing Change taxonomy. We collected respondent demographic information, facility-level characteristics, and case review completion rates (percentage of high-risk patients who received a case review). We used Kruskal-Wallis tests and negative binomial regression to assess strategy use and factors associated with case reviews.

**Results:**

Contacts at 89 of 140 facilities completed the survey (64%) and reported using a median of 23 (IQR 16–31) strategies. The median case review completion rate was 71% (IQR 48–95%). Neither the number or types of strategies nor completion rates differed by randomization arm. The most common strategies were using the STORM dashboard (97%), working with local opinion leaders (80%), and recruiting local partners (80%). Characteristics associated with case review completion rates included respondents being ≤ 35 years old (incidence rate ratio, IRR 1.35, 95% CI 1.09–1.67) and having < 5 years in their primary role (IRR 1.23; 95% CI 1.01–1.51), and facilities having more prior academic detailing around pain and opioid safety (IRR 1.40, 95% CI 1.12–1.75). Controlling for these characteristics, implementation strategies associated with higher completion rates included (1) monitoring and adjusting practices (adjusted IRR (AIRR) 1.40, 95% CI 1.11–1.77), (2) identifying adaptations while maintaining core components (AIRR 1.28, 95% CI 1.03–1.60), (3) conducting initial training (AIRR 1.16, 95% CI 1.02–1.50), and (4) regularly sharing lessons learned (AIRR 1.32, 95% CI 1.09–1.59).

**Conclusions:**

In this national evaluation of strategies used to implement case reviews of patients at high risk of opioid-related adverse events, point of contact age and tenure in the current role, prior pain-related academic detailing at the facility, and four specific implementation strategies were associated with case review completion rates, while randomization to additional centralized oversight was not.

**Trial registration:**

This project is registered at the ISRCTN Registry with number ISRCTN16012111. The trial was first registered on May 3, 2017.

Contributions to the literature

- There are many implementation strategies that sites can use to increase adoption of an evidence-based practice or program. However, not all strategies work well in all contexts.
- We found that contextual factors related to implementation champions and individual sites and 5 specific implementation strategies were important to successful implementation of reviewing opioid use among high-risk patients, whereas the total number of strategies used or a top-down mandate to implement the practice was not.
- These findings help to inform the implementation science literature about how and when different implementation strategies work in the real world.

## Background

Concerns about opioid misuse and overdose have led to substantial efforts to decrease high-risk opioid prescribing and improve opioid safety [1–7]. The Veterans Health Administration (VHA) has been on the forefront of opioid risk assessment and implementation of risk-mitigation strategies, such as the VA Opioid Safety Initiative, which has resulted in a rapid decrease in opioid prescribing—primarily decreasing initiation of long-term use—among Veterans who use VHA [4–8]. As we increase our understanding of the factors associated with adverse opioid-related events among patients prescribed opioids, we can identify sub-groups of patients who may benefit from further assessment and intervention.

As a part of a concerted national effort to identify patients prescribed opioids who are at high risk for adverse events, VA developed the Stratification Tool for Opioid Risk Mitigation (STORM), which uses a predictive model to incorporate electronic health record (EHR)-derived risk factors into a summary score that predicts risk of overdose- or suicide-related health care events or death in patients prescribed opioid analgesics [6, 9]. The VA Office of Mental Health and Suicide Prevention (OMHSP) then developed a suite of web-based reports to convey relevant information to support effective risk management strategies for Veterans at high risk of adverse events, subsequently referred to in this paper as the “STORM dashboard.” In April 2018, the VA issued Notice 2018-08 (updated the next year to Notice 2019-15) [10] requiring VA Medical Centers (“facilities”) to complete case reviews for Veterans prescribed opioids that the STORM risk model identified as being at very high risk for adverse outcomes. Case reviews involved using the STORM dashboard, chart review, or other data-based procedures to evaluate each patient’s risk level and to determine whether additional risk mitigation strategies (e.g., referral to a pain specialist, prescription of naloxone) were indicated. To examine the impact of mandated centralized oversight of implementation completion, half of the facilities were randomly assigned to receive a version of Notice 2018-08 that included language indicating that additional oversight—including the requirement of local action plans and receipt of external support—if facility case review completion rates (the percent of very high risk patients receiving a case review) were < 97% 6 months following the notice [9]. Our evaluation team was tasked with understanding how facilities responded to these policy notices in terms of implementation activities and case review completion rate [11].

We focused our evaluation on facility-level implementation strategies, i.e., specific methods or techniques used to enhance the adoption and sustainment of an evidence-based intervention or practice [12]. Without such strategies, the adoption and successful implementation of new treatments or processes often fail. The Expert Recommendations for Implementing Change (ERIC) group recently specified, defined, and clustered 73 implementation strategies [13–15]. We previously developed a tool to assess ERIC-defined implementation strategies in the context of VA’s national effort to expand the use of newer, more effective hepatitis C (HCV) medications [16, 17]. In these prior efforts, we found that the number of strategies used was associated with more treatment starts and that specific strategies were associated with improved uptake of the newer evidence-based medications. However, it was unclear whether this survey could inform other implementation efforts.

To understand how facilities responded to the policy notice in this national randomized program evaluation, we aimed to (1) assess implementation strategies that facilities used to implement case reviews, (2) determine whether those strategies differed for facilities receiving policy notices including additional oversight from the VA national office providing implementation support, and (3) examine the association between implementation strategies, respondent characteristics, facility characteristics, and case review completion rates.

## Methods

### Regulatory approval

The VA Pittsburgh Healthcare System Institutional Review Board approved this project as research. The protocol for this implementation evaluation was published previously [11].

### Survey development

Facilities were randomized to receive one of two versions of a policy notice that had the same specified goal but varied in the oversight they received. The survey was designed to identify how facilities responded to these notices and to characterize the implementation strategies they used. To measure the implementation strategies that facilities chose, the evaluation team adapted our prior implementation strategy survey to this evaluation [16, 17]. After review, four financial strategies were deemed irrelevant in VA and omitted from the survey (“capitated payments,” “change liability laws,” “place innovation on fee for service lists/formularies,” and “alter patient/consumer fees”). Furthermore, “develop and organize quality monitoring systems” was omitted because it was inherent to the STORM dashboard. Relevant examples were added to the survey as parenthetical statements where this was deemed necessary for clarity. The remaining 68 items were reviewed by partners at OMHSP, and minor edits were made to improve the clarity of the survey (Additional file 1). The strategies were arranged in the survey in 9 previously defined implementation clusters [14] and respondents were asked (1) whether facilities used each strategy to promote case review completion (yes/no) and (2) whether this strategy was used in response to the policy notice or was otherwise used at the facility. The latter question was included because opioid safety is an ongoing national VA priority prior to and including the time of the study, and some facilities may have instituted practices to improve opioid safety prior to or independent of Notice 2018-08. The survey also assessed the following demographic questions of participants: sex, gender, age, race/ethnicity, degree, professional role, hours spent in patient care per week, and opioid prescribing privileges (yes/no).

### Participant recruitment

OMHSP provided the email addresses for a point of contact responsible for implementing the policy notice at each facility. Of 140 facilities, 136 had a designated point of contact. We emailed initial survey invitations along with an electronic survey to primary points of contact at the end of Fiscal Year 2019, 6 months after the policy notice was released. We sent up to two weekly reminder emails followed by phone calls and instant messages through the VA communication system as necessary. If a primary point of contact did not respond and a secondary point of contact was on record, the secondary point of contact was contacted (*n* = 10). If a point of contact suggested a colleague who would be better suited to complete the interview, we contacted that colleague (*n* = 5).

### Case review completion rates

Case review completion rates for each facility were calculated as the number of case reviews that were completed for very high-risk patients (numerator) over the number of patients in the very high-risk category (denominator) between July to September 2018 (the fourth quarter of the VA fiscal year) for each facility. These data were available on a dashboard used by OMHSP to report a wide range of quality improvement metrics. A link to this dashboard and the case review metrics were prominently posted on the STORM dashboards. Our partners granted us access to the data to contribute to quality improvement efforts in VA.

### Facility-level covariates

The randomization arm was included as the primary independent variable. Sites were coded as receiving a policy notice with versus without language requiring additional oversight if the target case review completion rate was not achieved. We examined several facility characteristics described in detail in our published evaluation protocol [11]. In brief, we examined organizational factors following Glasgow et al.’s analytic framework, which includes two broad dimensions: (1) facility structure variables (e.g., rurality) and (2) staffing/culture variables (e.g., psychological safety). We focused on data available across VA facilities and those that stakeholders defined as most relevant to the implementation effort [18]. The “total number of outpatient visits per year” included all visits at the facility and was categorized into quartiles. Primary care panel size was defined as the number of primary care doctors per 100 patients. Facility complexity is a long-standing VA variable about the nature of the services provided at VA facilities. The score is classified numerically from 1 to 3, with level 1 being the most complex. Complexity is scored using national data on workload (patient load and acuity), research dollars, the availability of complex clinical programs (e.g., ICU care, transplant, neurosurgery), and location (i.e., rurality) [19]. As per prior work, we classified facilities as level 1 (higher level) vs. other [19]. Rurality was classified as yes/no, using the VA definition of rurality, which is based on the Rural-Urban Commuting Areas System and defines urban as “census tracts with at last 30% of the population residing in an urbanized area” [20, 21]. Because a facility’s case review completion rate could be higher if fewer case reviews were required, we included each facility’s number of patients in the “very high-risk” category as a covariate, defined as the lowest quartile of case reviews vs. other quartiles at baseline. “Workplace performance” was derived from employee ratings of 6 items from the 2018 VA All-Employee Survey that assessed resource availability, training, goals, and innovation [22]. This continuous score ranges from 1 to 5, with higher scores being more favorable.

VA’s Academic Detailing Service provides targeted one-on-one training and problem-solving, normative feedback, and educational materials for providers around high-priority practice improvement and prescribing challenges [6, 23–25]. We included the number of academic detailing events on relevant topics that occurred at each facility in the 6 months prior to the release of the policy notice, using Academic Detailing Service data provided by VA’s Pharmacy Benefits Management Services. Specifically, this included the number of academic detailing events that covered the STORM dashboard, pain dashboard, opioid use disorder, or other pain management topics. For analyses, we included a binary variable indicating whether facilities had any academic detailing events specifically around the STORM and/or other opioid safety dashboards (yes/no). We also categorized the overall number of pain-related academic detailing events into quartiles for analyses.

### Analyses

We compared facility characteristics between responding and non-responding facilities using *t* tests for continuous variables and chi-square tests for categorical variables and summarized the self-reported characteristics of responding points of contact using means and proportions, as appropriate. We computed a cross tabulation of each of the 68 implementation strategies overall and by randomization arm (policy notice type). We then computed the proportion of strategies used within each implementation strategy cluster [14] and the total number of strategies used overall and by randomization arm. We repeated this process to describe the number and type of strategies that were implemented as a direct response to the policy notice. We then assessed whether there were differences between the number and types of strategies used by randomization arm using Kruskal-Wallis tests.

We used negative binomial regression to model the case review completion rates with results reported as incident rate ratios (IRR). We first assessed the unadjusted associations between case review completion rates and the respondent and facility characteristics. Characteristics associated with the case review completion rates at the *p* < 0.15 level were included in subsequent adjusted analyses. We then modeled both the adjusted and unadjusted associations between each individual implementation strategy and the total number of strategies used and the case review completion rates. All analyses were conducted using SAS 9.4 (Cary, NC) and Stata 14 (College Station, TX).

## Results

### Respondents

A STORM point of contact from 101 facilities opened the survey, and 92 facilities completed surveys. Of these 92 facilities, case review completion rates were available for 89, and these 89 were included in the subsequent analyses. Responding facilities (*n* = 92) did not significantly differ from non-responding facilities in terms of facility-level characteristics (Additional file 2). However, there was a non-significant trend towards responding facilities being more likely than non-responding facilities to be level 1 complexity (72% vs. 54%, *p* = 0.07). Most responding points of contact were non-Hispanic and White (71%). Clinicians and pharmacists made up 83% of the sample, and 42% of points of contact reported that they had opioid prescribing privileges (Table 1).

**Table 1:** Characteristics of participants and facilities responding to ERIC survey

Characteristics of Participants and Facilities	*N* = 89
Participant characteristics	
Current gender identity (How do you describe yourself?), *n* (%)^b^	
Male	38 (43)
Female	47 (53)
Age, *n* (%)	
≤ 25 years	0 (0)
26–35 years	23 (26)
36–45 years	26 (29)
46–55 years	16 (18)
56 and older	20 (22)
Race ethnicity, *n* (%)^b^	
Non-Hispanic Asian	7 (8)
Non-Hispanic Black or African-American	5 (6)
Non-Hispanic White	63 (71)
Other race/ethnicity^a^	5 (6)
Highest degree, *n* (%)^b^	
Bachelor’s degree	4 (4)
Master’s degree	6 (7)
MD or DO	31 (35)
PharmD	36 (40)
Other doctoral degree	10 (11)
Years in primary role, *n* (%)	
5 years or less	38 (43)
6–10 years	18 (20)
11–15 years	16 (18)
16 years or more	14 (16)
What is your primary role in the VA? *n* (%)^b^	36 (40)
Administrator	12 (13)
Clinician	36 (40)
Pharmacist	37 (42)
Other	2 (2)
How many hours per week do you see patients? *n* (%)^b^	
≤ 10 h	31 (35)
11–20 h	14 (16)
21–30 h	21 (24)
31–40 h	19 (21)
Opioid prescribing privileges, *n* (%)^b^	37 (42)
**Facility characteristics**	
Number of visits, median (IQR)	561098 (195920,926276)
Facility complexity, *n* (%)	
1a, 1b, or 1c	66 (74)
2	8 (9)
3	15 (17)
Rural, *n* (%)	12 (14)
Case reviews requested, median (IQR)	18 (19)
PC panel patient size (per 100 patients), median (IQR)	918 (121)
Academic detailing through pain campaign, median (IQR)	21 (48)
Academic detailing around STORM dashboard, *n* (%)	48 (54)
All employee survey, workplace performance, median (IQR)	4 (0)

^a^Other includes those who indicated Hispanic ethnicity or more than one racial/ethnic group

^b^Percentages may not sum to 100% due to non-response/missing data; Proportion of participants who selected the option “prefer not to answer” for the following demographic questions: “current gender identity (4.5%),” “race ethnicity (10.1%),” “highest degree (2.3%),” “primary role in VA (2.3%),” “hours seeing patients (4.5%),” and “opioid prescribing privileges (2.3%)”

### Implementation strategy use

**Fig. 1:**
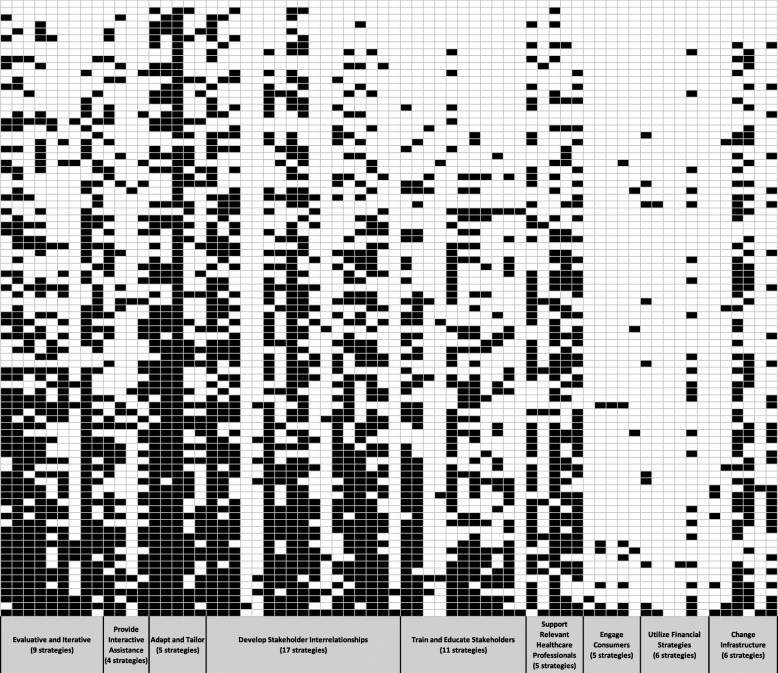
Strategy heat map. This figure shows the use of individual strategies, grouped by cluster, in order from first to last on the survey, for individual facilities. Each facility is a row, and the rows are sorted such that the facilities using the most strategies are at the bottom of the graphic. The box is black if a facility reported using the strategy and white if the facility did not report using the strategy

**Fig. 2:**
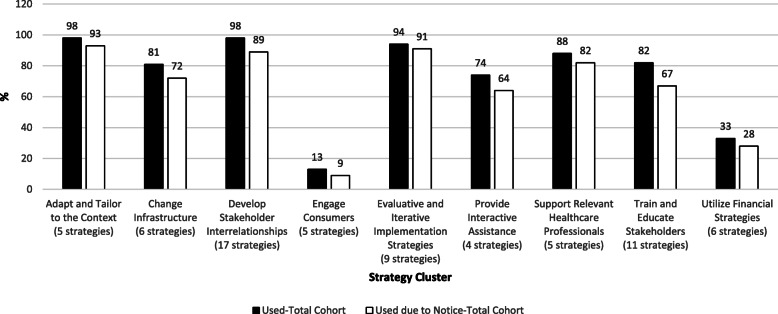
Implementation Strategy use by cluster. This figure illustrates use of strategies by cluster. The black bars illustrate the proportion of all facilities that used at least one strategy in a cluster. The white bars illustrate the proportion of facilities that reported using at least one strategy in the cluster because of the policy Notice

Included facilities (*n* = 89) endorsed a median of 23 (IQR 16–31) strategies and a median of 18 (IQR 11–25) strategies used specifically because of the policy notice. The most commonly used strategies were using the STORM dashboard (97%), informing local opinion leaders of the need to complete case reviews (80%), and recruiting and cultivating relationships with local partners (80%) (Additional file 3). Overall, facilities reported that most implementation strategies (80%) were attributed to the policy notice. Figure 1 illustrates the use of individual strategies, grouped by cluster and shown in order from first to last on the survey, for individual facilities. The most commonly used implementation clusters were “adapt and tailor to the context,” “develop stakeholder interrelationships,” and “evaluative and iterative strategies.” Ninety-four to 98% of facilities reported using at least one strategy in these clusters. The least commonly used cluster of strategies was “engage consumers” (i.e., patients); only 13% of facilities used any strategies from this cluster (Fig. 2).

There were no significant differences in the total number of implementation strategies used for the oversight vs. non-oversight randomization arm (median 25 vs. 23, respectively, *p* = 0.80). Although the proportion of facilities that used at least one strategy in a given implementation cluster did not vary by arm, facilities that were randomized to the oversight arm used numerically more strategies from the “adapt and tailor” cluster than facilities in the non-oversight arm (median of 4 vs. 3 strategies in the cluster, *p* = 0.049) (Table 2).

**Table 2:** Number of strategies used by cluster, overall and by randomization arm

Strategy cluster name (number of strategies in cluster)	Used				Used due to notice			
	Overall	Oversight	No oversight	*p* value	Overall	Oversight	No oversight	*p* value
Adapt and tailor (5 strategies)	**3.0 (1.5)**	**4.0 (1.0)**	**3.0 (2.0)**	**0.049**	3.0 (1.5)	3.0 (1.5)	3.0 (2.0)	0.56
Change infrastructure (6 strategies)	1.0 (1.0)	1.0 (1.0)	1.5 (1.0)	0.48	1.0 (2.0)	1.0 (2.0)	1.0 (1.5)	0.35
Develop stakeholder interrelationships (17 strategies)	7.0 (5.0)	7.0 (5.0)	7.0 (5.0)	0.74	6.0 (6.0)	6.0 (5.0)	6.0 (5.5)	0.93
Engage consumers (5 strategies)	0.0 (0.0)	0.0 (0.0)	0.0 (0.0)	0.97	0.0 (0.0)	0.0 (0.0)	0.0 (0.0)	0.93
Evaluative and iterative (9 strategies)	4.0 (4.0)	4.0 (4.0)	4.0 (4.0)	0.46	4.0 (3.0)	4.0 (3.0)	4.0 (4.0)	0.99
Provide interactive assistance (4 strategies)	1.0 (2.0)	1.0 (2.0)	1.0 (1.0)	0.74	1.0 (2.0)	1.0 (1.0)	1.0 (2.0)	0.29
Support relevant healthcare professionals (5 strategies)	2.0 (2.0)	2.0 (3.0)	3.0 (1.5)	0.84	2.0 (2.0)	2.0 (2.0)	2.0 (2.0)	0.37
Train and educate stakeholders (11 strategies)	3.0 (4.0)	3.0 (4.0)	3.0 (4.0)	0.56	2.0 (3.0)	1.0 (3.0)	2.5 (3.5)	0.07
Utilize financial strategies (6 strategies)	0.0 (1.0)	0.0 (1.0)	0.0 (1.0)	0.57	0.0 (1.0)	0.0 (0.0)	0.0 (1.0)	0.50
**Total number of strategies (median, IQR)**	23.0 (15.0)	25.0 (13.0)	23.0 (15.5)	0.80	18.0 (14.0)	18.0 (13.0)	18.0 (17.5)	0.56

*Statistically significant relationships are highlighted in bold; median (IQR) presented

### Case review completion rates

The median number of case reviews requested of the facilities at baseline was 18 (IQR 9–28). The median case review completion rate was 71% (IQR 48–95%). Overall, 18 facilities (20%) met the 97% target completion rate established a priori by OMHSP. Case review completion rates did not significantly differ by randomization arm (78% in the non-oversight arm vs. 71% in the additional oversight arm, *p* = 0.26). However, the non-oversight randomization arm was significantly more likely to meet the 97% target (30% vs. 11%, *p* = 0.04).

In univariate analyses (Table 3), respondent characteristics associated with higher case review completion rates included younger age (incidence rate ratio, IRR 1.35, 95% CI 1.09–1.67 for age ≤ 35 vs. older) and having less than 5 years in their current primary role (IRR 1.23; 95% CI 1.01–1.51). Only one facility characteristic, increased exposure to pain-related academic detailing prior to baseline, was associated with higher case review completion rates (IRR 1.40, 95% CI 1.12–1.75).

**Table 3:** Factors associated with case review completion rate

Characteristic	IRR (CI)	***p*** value
**Respondent characteristics**		
Female sex	1.21 (0.99–1.49)	0.07
Age		
26–35 years	–	**0.01**
36–45 years	0.63 (0.49–0.82)	
46–55 years	0.81 (0.63–1.06)	
56 years and older	0.79 (0.70–0.99)	
Age > 35 years vs ≤ 35 years	**0.74 (0.60**–**0.92)**	**0.007**
Race/ethnicity		0.98
Non-Hispanic White	–	
Non-Hispanic Asian	0.94 (0.64–1.39)	
Non-Hispanic Black	0.92 (0.60–1.41)	
Other race/ethnicity	1.02 (0.67–1.54)	
Highest degree		0.72
Bachelor’s degree	0.77 (0.46–1.31)	
Master’s degree	1.01 (0.66–1.54)	
MD or DO	0.87 (0.70–1.10)	
Other doctoral degree	0.89 (0.64–1.23)	
PharmD	–	
Years in primary role > 5 years	**0.81 (0.66**–**0.99)**	**0.04**
What is your primary role in the VA?		0.55
Administrator	0.85 (0.62–1.18)	
Clinician	0.87 (0.70–1.08)	
Pharmacist	–	
Other	0.79 (0.33–1.92)	
How many hours per week do you see patients?*		0.16
≤ 10 h	–	
11–20 h	1.19 (0.88–1.60)	
21–30 h	0.81 (0.62–1.06)	
31–40 h	0.98 (0.76–1.28)	
Has opioid prescribing privileges	0.94 (0.77–1.16)	0.57
**Facility structure**		
Number of visits		0.94
1st quartile	–	
2nd quartile	1.09 (0.79–1.50)	
3rd quartile	1.06 (0.78–1.43)	
4th quartile	1.10 (0.81–1.48)	
Facility complexity		0.90
2 (vs. 1)	0.90 (0.58–1.41)	
3 (vs. 1)	1.00 (0.74–1.35)	
Rural	1.03 (0.73–1.46)	0.87
Case reviews requested (1st quartile vs. others)	0.85 (0.62–1.16)	0.30
**Staffing/culture**		
PC panel patient size (per 100 patients)	0.94 (0.85–1.03)	0.19
Academic detailing through the pain campaign	**1.40 (1.12**–**1.75)**	**0.003**
Academic detailing around STORM dashboard	0.91 (0.75–1.11)	0.37
Workplace performance (per .1)	1.00 (0.91–1.10)	0.95

Statistically significant relationships are highlighted in bold

^a^Six respondents reported that they did not see patients. and were classified as ≤ 10 h

Controlling for these characteristics, the implementation strategies associated with higher case review completion rates (Table 4) were (1) “regular monitoring and adjusting practices as needed” (adjusted IRR, AIRR 1.40, 95% CI 1.11–1.77), (2) “identifying ways that the process of completing case reviews of very high-risk patients can be adapted to meet local needs while still maintaining the core components of the review process” (AIRR 1.28, 95% CI 1.03–1.60), and two education strategies: (3) “conducting an initial training session” (AIRR 1.16, 95% CI 1.02–1.50) and (4) “creating or participating in groups that meet regularly to discuss and share lessons learned” (AIRR 1.32, 95% CI 1.09–1.59).

**Table 4:** Associations between implementation strategies and case review completion rates

	Unadjusted		Adjusted	
Strategy number, cluster, and name	IRR (95% CI)	*P* value	IRR (95% CI)	*P* value
S5 Evaluate—develop a written implementation plan including goals and strategies	0.84 (0.68–1.04)	0.11	0.82 (0.67–1.01)	0.06
S8 Evaluate—regular monitoring and adjusting practices (as needed) for completing mandated case reviews of very high-risk patients	**1.29 (1.04**–**1.62)**	**0.02**	**1.40 (1.11**–**1.77)**	**0.004**
S11 Interactive assistance—use a centralized system, for example from the VISN, to deliver technical assistance	0.87 (0.70–1.07)	0.19	0.85 (0.69–1.04)	0.11
S14 Adapt and tailor—identify ways that the process of completing case reviews of very high-risk patients can be adapted to meet local needs while still maintaining the core components of the review process	**1.29 (1.03**–**1.62)**	**0.03**	**1.28 (1.03**–**1.60)**	**0.03**
S29 Stakeholder interrelationships—obtain formal written commitments from key local stakeholders that state what they will do to support the completion of mandated case reviews, for example, written agreements with CBOCs or between service lines	0.63 (0.40–1.00)	0.05	0.66 (0.43–1.01)	0.06
S36 Train/educate—conduct an initial training session	1.16 (0.95–1.41)	0.15	**1.23 (1.02**–**1.50)**	**0.03**
S40 Train/educate—create or participate in groups that meet regularly to discuss and share lessons learned	**1.26 (1.03**–**1.52)**	**0.02**	**1.32 (1.09**–**1.59)**	**0.004**
S45 Train/educate—train designated relevant healthcare professionals at your medical center to train others to complete mandated case reviews	1.21 (0.98–1.50)	0.07	1.22 (1.00–1.49)	0.06

*Statistically significant associations are in bold

## Discussion

We found that facility contacts used diverse implementation strategies in response to a policy notice requiring them to complete case reviews for patients prescribed opioids who were estimated to be at high risk for adverse events. Randomization to being required to receive additional centralized oversight if a facility failed to meet an a priori target did not significantly impact the implementation strategies that facilities chose. However, we did identify several respondent and facility characteristics and implementation strategies that were associated with improved case review implementation in this national opioid safety effort.

While the average facility used 23 implementation strategies, we found that only a few key strategies were associated with case review completion rates. Education and adaptation/tailoring emerged as important implementation strategies in the adjusted models. Specific strategies associated with increased case review completion rates included adjusting practices based on regular monitoring and adapting practices as needed, while retaining fidelity to critical components of the implementation effort. The ability to adapt/tailor the efforts to local needs is generally considered to be important in other implementation efforts [26, 27], and leadership supported and encouraged adaptation in the STORM implementation effort. Understanding which implementation strategies work when and in what context is the “holy grail” of implementation science and can enhance the efficiency, cost-effectiveness, and effectiveness of implementation more broadly.

In addition to the specific survey-defined strategies that were actively used during implementation, we found that pre-implementation academic detailing was one of the strongest predictors of case review completion. Academic detailing is, in and of itself, an evidence-based, multi-component implementation strategy that includes needs assessments, education, and focused training and has contributed to successful implementation efforts across a number of domains [24, 28–31]. In VA, academic detailing is typically conducted by clinical pharmacists, who deliver face-to-face, 1-on-1 training. Our findings support the notion that academic detailing is effective as a pre-implementation, or “preparation” strategy, as we found that academic detailing measured prior to implementation was associated with the outcome. Engaging in academic detailing may also reflect other site-level contextual factors such as engagement, enthusiasm, or leadership support.

While relatively few implementation strategies were important in predicting case review completion rates, two respondent characteristics were significantly associated with this outcome. These included younger age of the respondent and fewer years in one’s current VA role. This finding is consistent with other studies that have also found that younger and more recently trained clinicians are more likely to be early adopters of innovations [32]. Alternatively, this association could be the result of an unmeasured confounder. Future work should assess how team member characteristics relate to use of specific implementation strategies and implementation success.

We notably found that randomization to policy notices requiring additional oversight did not positively influence facility case review completion rates. The two notices differed in that the “additional oversight” notices included language that a site would be required to receive additional oversight and support if it did not reach the 97% case review completion. One possible explanation for why the inclusion of this requirement did not affect implementation or case review completion is that we measured implementation strategies and case review completion rates too soon after the policy notice was released and that all sites needed more time to stand up effective processes to complete the case reviews. A second possibility is that the differences in the policy notices were too minor to make a positive impact. A third possibility is that the requirement of “additional oversight” was in fact detrimental to implementation success. Though this was possible by chance alone, the facilities randomized to the “additional oversight” policy notice were less likely to reach the 97% threshold of case review completion rates than those with the standard policy notice. If points of contact perceived “additional oversight” to be a threat or negative consequence, the effect may have been detrimental, in contrast to a potential notice that included a positive incentive or reward. This is a well-established psychological phenomenon wherein positive reinforcement leads to increased intrinsic motivation more so than negative consequences [33]. Another complicating factor is that VA has an additional layer of regional management between health care systems and the national office that would provide additional oversight per the notice. Although regional management was made aware that the notice only required oversight and action planning at a randomized subset of sites per the notice, some chose to globally implement their own oversight and action planning requirements across sites in their region, per their standard practices. Variable regional oversight practices may have minimized effects of centralized national oversight.

We found several key similarities and differences when we compared our results from this survey to previously published results from a similar survey conducted in the context of VA’s national HCV elimination program. In both implementation efforts, respondents endorsed a similarly high number of strategies, with a median number of 23 vs. 24 implementation strategies in the STORM and HCV efforts, respectively. We also identified strategies associated with more successful implementation that were common to both efforts, including training/education and tailoring to the context [16, 17]. Tailoring the survey for the STORM implementation effort allowed us to reduce the number of strategies that we assessed (from 73 to 68). That we found useful information from both assessments of stakeholder-reported implementation strategies in vastly different implementation efforts speaks to the value of this approach to assessing implementation strategies. An overarching goal in the field of implementation science is to systematize how investigators and healthcare systems choose implementation strategies to address implementation barriers. Moving forward, we hope to develop data around which implementation strategies function best in which settings to address which implementation goals. Then, we can test the application of these strategies using randomized experiments or other large, naturalistic, pragmatic operational initiatives like STORM.

There were also key differences between the national opioid case review and HCV treatment implementation efforts. First, few facilities reported engaging patients in efforts to implement case reviews for very high-risk patients, since this effort was focused on a provider activity. This is in contrast to the HCV effort, where patient-facing strategies were universally used and associated with increased treatment [16, 17]. In the HCV effort, the characteristics of the individual respondents were not associated with the outcomes of interest, while the point of contact demographic characteristics appeared to be important to opioid-related case review completion. These key differences may be explained by the differences between the implementation “ask” in these two efforts. The case review effort could be completed by a single team, since the average facility had 18 very high-risk patients. In contrast, in HCV treatment implementation, facilities were asked to treat hundreds of patients, which may have required coordinated implementation efforts across a range of stakeholders. This demonstrates the importance of understanding the context and the “ask,” or complexity, of an implementation effort when determining which strategies to use and how to interpret findings. Stemming from Rogers’ work on diffusion of innovations and incorporated into leading implementation frameworks like the Consolidated Framework for Implementation Research, there is good evidence to show that the complexity of the innovation impacts implementation success [34, 35]. Complexity also likely impacts the choice of implementation strategies. Measuring and evaluating the linkages between complexity, innovation implementation, and implementation strategies could inform how to choose implementation strategies based on the complexity of the innovation so as to improve implementation outcomes.

We acknowledge several notable limitations of this study. First, implementation strategies were reported by a single individual from each facility and may not have reflected the full scope of what was being done at the facility level. However, we have previously found high interrater reliability between multiple respondents from the same facility in a similar study using a similar survey [17]. An additional limitation is the potential for contamination across the randomization arms and unblinding to the process of randomization. While facilities were not made aware that two different policy notices were assigned randomly, it is possible that providers could have communicated and become aware of the randomization process, which may have altered the implementation strategies that were chosen. While the survey provides information about whether facilities used a wide range of strategies, it does not address other key elements about the strategies (e.g., intensity, mechanism, fidelity to the strategy), so a key next step is to collect data that better elucidates how the strategies are used. Another limitation is that respondents may not cognitively distinguish the strategies employed at a particular facility. In addition, they may not appreciate how the implemented strategies were defined or whether the strategies and clusters were useful. We tried to mitigate this limitation by engaging stakeholders in survey development, adding examples relevant to the clinical domain, and clustering the strategies allowed respondents to reach the end of the survey. Nevertheless, future studies could examine whether there were respondent issues and whether our strategies to overcome this limitation are effective. Finally, we conducted multiple statistical tests using a relatively small number of facilities, which allows us to generate hypotheses but not draw definitive conclusions from the findings. Despite these limitations, this was a national, randomized program evaluation with excellent response rates, and our findings add to a growing body of literature assessing a wide variety of implementation strategies across large-scale implementation efforts.

## Conclusions

In conclusion, collecting implementation strategy data in this national randomized program evaluation allowed us to track and compare implementation activities across randomization arms and assess their associations with a meaningful implementation metric: case review completion rates. These findings add to the growing body of literature addressing the measurement and interpretation of implementation strategy data. We found that facilities with more pre-implementation academic detailing and younger implementation points of contact who were more recent to their current role, in addition to facilities using education, adaptation, and tailoring strategies, were more successful in implementing case reviews on very high-risk patients.

## Supplementary information

**Additional file 1.** Implementation strategy survey.

**Additional file 2.** Characteristics of participating vs. non-participating facilities.

**Additional file 3.** Use of implementation strategies: overall and due to the policy notice, by randomization arm (*n*=89).

## Data Availability

All data generated or analyzed during this study are included in this published article.

## Abbreviations

ERIC: Expert Recommendations for Implementing Change
FY18: Fiscal Year 2018
HCV: Hepatitis C virus
IIR: Incident rate ratios
OMHSP: Office of Mental Health and Suicide Prevention
STORM: Stratification Tool for Opioid Risk Mitigation
VA: Veterans Affairs
VHA: Veterans Health Administration
