# Supplementary material for: Tracking implementation strategies in the randomized rollout of a Veterans Affairs national opioid risk management initiative
Source: Implement Sci. 2020 Jun 23;15:48. doi: 10.1186/s13012-020-01005-y (PMC7313133; doi:10.1186/s13012-020-01005-y)
Supplement: Supplementary file 2 — Additional file 2. Characteristics of participating vs. non-participating facilities. [file 13012_2020_1005_MOESM2_ESM.docx]

**Additional file 2. Characteristics of Participating vs. Non-Participating Sites**

| **Facility Characteristics** | **Overall**  **(N=140)** | **Completed Survey**  **(N=92)** | **Did not complete Survey**  **(N=48)** | **P-Value** |
| --- | --- | --- | --- | --- |
| Complexity, n (%) |  |  |  | 0.0667 |
| 1a, 1b, or 1c | 92 (66) | 66 (72) | 26 (54) |  |
| 2 | 20 (14) | 9 (10) | 11 (23) |  |
| 3 | 28 (20) | 17 (19) | 11 (23) |  |
| Urban Rural, n (%) |  |  |  | 0.7883 |
| Rural | 17 (12) | 12(13) | 5 (10) |  |
| Urban | 123 (88) | 80(87) | 43 (90) |  |
| Adjusted Primary Care Patient Panel Size, median (IQR) | 921.6 (129) | 919.7 (118) | 924.4 (148) | 0.7842 |
| Workplace performance, median (IQR) | 3.8 (0) | 3.8 (0) | 3.8 (0) | 0.9860 |
