# Supplementary material for: Tracking implementation strategies in the randomized rollout of a Veterans Affairs national opioid risk management initiative
Source: Implement Sci. 2020 Jun 23;15:48. doi: 10.1186/s13012-020-01005-y (PMC7313133; doi:10.1186/s13012-020-01005-y)
Supplement: Supplementary file 3 — Additional file 3. Use of implementation strategies: overall and due to the policy notice, by randomization arm (n=89). [file 13012_2020_1005_MOESM3_ESM.docx]

**Additional file 3. Use of Individual ERIC Strategies Overall and Due to the Policy Notice, by Facilitation Arm (n=89)**

| **Strategy** | **Used** | **Oversight**  **(n=45)** | **No Oversight**  **(n=44)** | **Used due to Notice** | **Oversight**  **(n=45)** | **No Oversight**  **(n=44)** |
| --- | --- | --- | --- | --- | --- | --- |
| **EVALUATIVE AND ITERATIVE** |  | | | | | |
| 1. Assess readiness to change | 49 (55) | 27 (60) | 22 (50) | 35 (39) | 18 (40) | 17 (39) |
| 2. Collect data on the completion of mandated case reviews of very high risk patients and use it to provide feedback and modify behavior | 61 (69) | 31 (69) | 30 (68) | 53 (60) | 23 (51) | 30 (68) |
| 3. Implement small changes in ways to improve completing mandated case reviews of very high risk patients, then assess those changes using performance data before making system-wide changes | 40 (45) | 19 (42) | 21 (48) | 36 (40) | 16 (36) | 20 (46) |
| 4. Collect and analyze information about the resources that your facility would need to complete mandated case reviews of very high risk patients | 54 (61) | 29 (64) | 25 (57) | 50 (56) | 26 (58) | 24 (55) |
| 5. Develop a written implementation plan including goals and strategies | 28 (32) | 16 (36) | 12 (27) | 23 (26) | 13 (29) | 10 (23) |
| 6. Develop and use local quality-monitoring systems that could include standards, protocols, or other tools | 37 (42) | 20 (44) | 17 (37) | 23 (26) | 13 (29) | 10 (23) |
| 7. Obtain and use patient or family feedback on having their case reviewed for opioid risk management | 10 (11) | 6 (13) | 4 (9) | 4 (5) | 2 (4) | 2 (5) |
| 8. Regular monitoring and adjusting practices (as needed) for completing mandated case reviews of very high risk patients | 62 (70) | 33 (73) | 29 (66) | 55 (62) | 29 (64) | 26 (59) |
| 9. Start with small pilot initiatives to increase the rate of completing mandated case reviews of very high risk patients and then scale them up | 42 (47) | 21 (47) | 21 (48) | 38 (43) | 20 (44) | 18 (41) |
| **INTERACTIVE ASSISTANCE** |  | | | | | |
| 10. Assign someone from the local medical center to help relevant healthcare professionals with the clinical issues involved in completing mandated case reviews of very high risk patients (sometimes called "technical assistance") | 46 (52) | 24 (53) | 22 (50) | 38 (43) | 20 (44) | 18 (41) |
| 11. Use a centralized system, for example from the VISN, to deliver technical assistance | 26 (29) | 12 (27) | 14 (32) | 21 (24) | 8 (18) | 13 (30) |
| 12. Use an outside facilitator to provide coaching, education, or other help with implementation | 8 (9) | 3 (7) | 5 (11) | 6 (7) | 1 (2) | 5 (11) |
| 13. Provide supervision on how to complete mandated case reviews of very high risk patients | 37 (42) | 19 (42) | 18 (41) | 30 (34) | 14 (31) | 16 (36) |
| **ADAPT AND TAILOR** |  | | | | | |
| 14. Identify ways that the process of completing case reviews of very high risk patients can be adapted to meet local needs while still maintaining the core components of the review process | 66 (74) | 36 (80) | 30 (68) | 59 (66) | 33 (73) | 26 (59) |
| 15. Tailor strategies to complete case reviews of very high risk patients to address specific barriers identified in your medical center | 62 (70) | 34 (76) | 28 (64) | 53 (60) | 28 (62) | 25 (57) |
| 16. Use the STORM dashboard to facilitate completion of mandated case reviews | 86 (97) | 43 (96) | 43 (98) | 81 (91) | 40 (89) | 41 (93) |
| 17. Use data from sources other than the STORM dashboard, for example OTTR or the Corporate Data Warehouse, to facilitate completion of mandated case reviews | 41 (46) | 24 (53) | 17 (39) | 26 (29) | 13 (29) | 13 (30) |
| 18. Consult data experts to help your medical center use or understand data about your patients who are prescribed opioids | 30 (34) | 17 (38) | 13 (30) | 17 (19) | 8 (18) | 9 (21) |
| **STAKEHOLDER INTERRELATIONSHIPS** |  | | | | | |
| 19. Recruit and cultivate relationships with local partners in your medical center to facilitate the completion of mandated case reviews | 71 (80) | 37 (82) | 34 (77) | 59 (66) | 31 (69) | 28 (64) |
| 20. Document and share with other medical centers knowledge gained from local efforts to complete mandated case reviews | 50 (56) | 26 (58) | 24 (55) | 37 (42) | 19 (42) | 18 (41) |
| 21. Include local relevant healthcare professionals and other stakeholders in discussions of whether conducting mandated case reviews is an appropriate method of opioid risk management | 54 (61) | 31 (69) | 23 (52) | 39 (44) | 22 (49) | 17 (39) |
| 22. Partner with a university to share ideas about completing case reviews of very high risk patients | 1 (1) | 1 (2) | 0 (0) | 0 (0) | 0 (0) | 0 (0) |
| 23. Develop and distribute a glossary of terms that relevant healthcare professionals might not be familiar with but are important for completing mandated case reviews | 6 (7) | 3 (7) | 3 (7) | 3 (3) | 0 (0) | 3 (7) |
| 24. Select or train local relevant healthcare professionals who will dedicate themselves to promoting the completion of mandated case reviews of very high risk patients | 60 (67) | 32 (71) | 28 (64) | 48 (54) | 25 (56) | 23 (52) |
| 25. Identify those who were quick to start case reviews once asked (early adopters) to learn from their experiences completing mandated case reviews | 29 (33) | 12 (27) | 17 (39) | 23 (26) | 11 (24) | 12 (27) |
| 26. Inform local opinion leaders about the need to complete case reviews of very high risk patients (e.g., clinical/administrative leaders or other influential colleagues) | 71 (80) | 36 (80) | 35 (80) | 66 (74) | 35 (78) | 31 (71) |
| 27. Involve the medical executive board or other existing governing structures in supporting progress towards the completion of mandated case reviews | 61 (69) | 34 (76) | 27 (61) | 53 (60) | 29 (64) | 24 (55) |
| 28. Simulate the completion of mandated case reviews to identify potential problems | 25 (28) | 14 (31) | 11 (25) | 21 (24) | 11 (24) | 10 (23) |
| 29. Obtain formal written commitments from key local stakeholders that state what they will do to support the completion of mandated case reviews, for example written agreements with CBOCs or between service lines | 5 (6) | 4 (9) | 1 (2) | 3 (3) | 2 (4) | 1 (2) |
| 30. Provide protected time to allow people to complete mandated case reviews to meet and share lessons | 40 (45) | 21 (47) | 19 (43) | 33 (37) | 18 (40) | 15 (34) |
| 31. Capitalize on existing high-quality networks to promote information sharing and problem solving related to implementing mandated case reviews | 47 (53) | 21 (47) | 26 (59) | 32 (36) | 12 (27) | 20 (46) |
| 32. Recruit, designate, or train leaders specifically to manage the completion of mandated case reviews at their medical center | 49 (55) | 24 (53) | 25 (57) | 48 (54) | 23 (51) | 25 (57) |
| 33. Seek input from advisory boards and interdisciplinary workgroups on ways to complete mandated case reviews of very high risk patients | 52 (58) | 26 (58) | 26 (59) | 41 (46) | 20 (44) | 21 (48) |
| 34. Seek the guidance of experts on implementing change in healthcare settings | 34 (38) | 15 (33) | 19 (43) | 22 (25) | 10 (22) | 12 (27) |
| 35. Visit sites outside your medical center that have been successful at completing mandated case reviews to learn from their experiences | 2 (2) | 1 (2) | 1 (2) | 2 (2) | 1 (2) | 1 (2) |
| **TRAIN AND EDUCATE** |  | | | | | |
| 36. Conduct an initial training session | 45 (51) | 21 (47) | 24 (55) | 36 (40) | 16 (36) | 20 (46) |
| 37. Provide ongoing training | 42 (47) | 18 (40) | 24 (55) | 33 (37) | 13 (29) | 20 (46) |
| 38. Have an outside expert in completing the mandated case reviews meet with relevant healthcare professionals to provide general trainings one-on-one or in groups | 7 (8) | 1 (2) | 6 (14) | 5 (6) | 0 (0) | 5 (11) |
| 39. Have an outside expert meet with relevant healthcare professionals to provide case-specific guidance and feedback | 4 (5) | 0 (0) | 4 (9) | 3 (3) | 0 (0) | 3 (7) |
| 40. Create or participate in groups that meet regularly to discuss and share lessons learned | 46 (52) | 25 (56) | 21 (48) | 27 (30) | 12 (27) | 15 (34) |
| 41. Develop local educational materials, like guidelines, manuals, or toolkits | 30 (34) | 16 (36) | 14 (32) | 20 (23) | 9 (20) | 11 (25) |
| 42. Distribute relevant educational materials | 41 (46) | 22 (49) | 19 (43) | 27 (30) | 12 (27) | 15 (34) |
| 43. Vary education or training methods to cater to different learning styles | 19 (21) | 9 (20) | 10 (23) | 11 (12) | 4 (9) | 7 (16) |
| 44. Give relevant healthcare professionals opportunities to shadow or otherwise observe experts conducting case reviews of very high risk patients | 21 (24) | 10 (22) | 11 (25) | 13 (15) | 4 (9) | 9 (21) |
| 45. Train designated relevant healthcare professionals at your medical center to train others to complete mandated case reviews | 26 (29) | 12 (27) | 14 (32) | 21 (24) | 9 (20) | 12 (27) |
| 46. Use educational institutions to train relevant healthcare professionals to complete mandated case reviews | 2 (2) | 1 (2) | 1 (2) | 1 (1) | 0 (0) | 1 (2) |
| **SUPPORT PROVIDERS** |  | | | | | |
| 47. Create new teams to conduct mandated case reviews of very high risk patients, for example interdisciplinary clinical workgroups | 53 (60) | 28 (62) | 25 (57) | 49 (55) | 25 (56) | 24 (55) |
| 48. Develop resource-sharing agreements with organizations or groups that have resources to help implement mandated case reviews | 10 (11) | 6 (13) | 4 (9) | 8 (9) | 4 (9) | 4 (9) |
| 49. Provide ongoing data to relevant healthcare professionals to facilitate the completion of mandated case reviews | 57 (64) | 24 (53) | 33 (75) | 47 (53) | 20 (44) | 27 (61) |
| 50. Develop reminders for relevant healthcare professionals to complete mandated case reviews of very high risk patients. Reminders could be delivered verbally, on paper, or electronically | 41 (46) | 19 (42) | 22 (50) | 34 (38) | 16 (36) | 18 (41) |
| 51. Revise professional roles to facilitate completion of mandated case reviews, including removing barriers or expanding roles, for example allow pharmacists to conduct the reviews | 47 (53) | 27 (60) | 20 (46) | 37 (42) | 18 (40) | 19 (43) |
| **ENGAGE CONSUMERS** |  | | | | | |
| 52. Encourage patients to prompt relevant healthcare professionals to complete mandated case reviews | 2 (2) | 0 (0) | 2 (5) | 2 (2) | 0 (0) | 2 (5) |
| 53. Intervene with patients or family members to promote acceptance of having their case reviewed | 6 (7) | 3 (7) | 3 (7) | 2 (2) | 0 (0) | 2 (5) |
| 54. Involve patients or family members in the effort to ensure that mandated case reviews are completed | 2 (2) | 1 (2) | 1 (2) | 1 (1) | 0 (0) | 1 (2) |
| 55. Engage in efforts to prepare patients to be active participants in getting their cases reviewed | 7 (8) | 3 (7) | 4 (9) | 3 (3) | 1 (2) | 2 (5) |
| 56. Use local announcements, newsletters, or other media strategies to reach large numbers of patients or relevant healthcare professionals about completing mandated case reviews of very high risk patients | 4 (5) | 3 (7) | 1 (2) | 4 (5) | 3 (7) | 1 (2) |
| **FINANCIAL** |  | | | | | |
| 57. Acquire new or use existing funding to facilitate the completion of mandated case reviews of very high risk patients | 10 (11) | 4 (9) | 6 (14) | 9 (10) | 4 (9) | 5 (11) |
| 58. Use financial incentives to promote completion of mandated case reviews | 2 (2) | 1 (2) | 1 (2) | 2 (2) | 1 (2) | 1 (2) |
| 59. Add new financial penalties for failure to complete mandated case reviews, for example reduce pay or deny standard bonuses | 0 (0) | 0 (0) | 0 (0) | 0 (0) | 0 (0) | 0 (0) |
| 60. Pursue new funding mechanisms, like grants or contracts, to support the completion of mandated case reviews | 1 (1) | 1 (2) | 0 (0) | 1 (1) | 1 (2) | 0 (0) |
| 61. Make it easier to account for and document workload involved in completing mandated case reviews | 23 (26) | 12 (27) | 11 (25) | 19 (21) | 9 (20) | 10 (23) |
| 62. Use payment-related approaches other than those already mentioned to motivate completion of mandated case reviews | 0 (0) | 0 (0) | 0 (0) | 0 (0) | 0 (0) | 0 (0) |
| **INFRASTRUCTURE** |  | | | | | |
| 63. Work with relevant accrediting bodies and professional membership organizations to encourage or require the use of case reviews of very high risk patients | 5 (6) | 2 (4) | 3 (7) | 4 (5) | 2 (4) | 2 (5) |
| 64. Change physical structure or availability of equipment to encourage completion of mandated case reviews | 5 (6) | 2 (4) | 3 (7) | 4 (5) | 2 (4) | 2 (5) |
| 65. Make change(s) to the standard STORM case-review note template in CPRS to make it easier to document the case reviews | 51 (57) | 26 (58) | 25 (57) | 45 (51) | 23 (51) | 22 (50) |
| 66. Change the location where mandated case reviews are completed, for example provide an interdisciplinary setting to complete case reviews | 42 (47) | 18 (40) | 24 (55) | 33 (37) | 13 (29) | 20 (46) |
| 67. Encourage an existing credentialing or licensing organization to certify relevant healthcare professionals in the completion of mandated case reviews or create a group with the authority to do so | 2 (2) | 1 (2) | 1 (2) | 2 (2) | 1 (2) | 1 (2) |
| 68. Have local leaders, like the facility director, department or service line leaders, state publicly that completing the mandated case reviews is a priority | 30 (34) | 15 (33) | 15 (34) | 26 (29) | 11 (24) | 15 (34) |
